# Harnessing chia seed polysaccharides for green synthesis of copper-based nanoparticles with antidiabetic and anti-inflammatory activities

**DOI:** 10.1371/journal.pone.0361692

**Published:** 2026-10-30

**Authors:** Farzana Khan, Yusra Jamil, Raeesa Wajid, Adnan Khan, Muhammad Yaseen, Douglas Law, Mostafa A. Abdel-Maksoud, Saeedah Almutairi, Wahidah H. Al-Qahtani, Waheed Ahmad, Patricio R. De los Ríos-Escalante, Ayaz Ahmad

**Affiliations:** 1 Department of Biotechnology, Abdul Wali Khan University Mardan, Khyber Pakhtunkhwa, Pakistan; 2 Faculty of Health and Life Sciences, INTI International University, Nilai, Negeri Sembilan, Malaysia; 3 Research Chair of Biomedical Applications of Nanomaterials, Biochemistry Department, College of Science, King Saud University, Saudi Arabia; 4 Botany and Microbiology Department, College of Science, King Saud University, Saudi Arabia; 5 Department of Food Sciences & Nutrition, College of Food and Agricultural Sciences, King Saud University, Riyadh, Saudi Arabia; 6 Minnesota Dental Research Center for Biomaterials and Biomechanics, School of Dentistry, University of Minnesota, Minneapolis, Minnesota, United States of America; 7 Departamento de Ciencias Biológicas y Químicas, Facultad de Recursos Naturales, Universidad Católica de Temuco, Chile; 8 Núcleo de Estudios Ambientales, Facultad de Recursos Naturales, Universidad Católica de Temuco, Chile; 9 Institute of Genetics and Developmental Biology, Chinese Academy of Sciences, Beijing, China; Yeungnam University, KOREA, REPUBLIC OF

## Abstract

Diabetes mellitus and chronic inflammation are global health issues, exacerbated by the incomplete control of metabolic-inflammatory pathways and the adverse effects of long-term pharmacotherapy. The development of multifunctional therapeutic nanomaterials via green nanotechnology has recently garnered attention. In this study, we synthesized copper-based nanoparticles (Cu NPs) using *Salvia hispanica* (chia) seed-derived polysaccharides and characterized their in vitro anti-inflammatory and antidiabetic activities, as well as in vivo antidiabetic effects in an alloxan-induced diabetic mouse model. Successful formation of polysaccharide-based Cu NPs was confirmed using UV-Visible, FTIR, XRD, and SEM analyses. Hemocompatibility assessment showed concentration-dependent hemolysis by Cu NPs (0.92 ± 0.06%, 5.71 ± 0.44%, and 8.42 ± 0.63% at the respective tested concentrations), indicating reduced hemocompatibility at higher concentrations. In the HRBC membrane stabilization assay, Cu NPs exhibited dose-dependent inhibition of hemolysis (45.83 ± 1.7% to 70.59 ± 2.1%), while heat hemolysis was reduced by 72.91 ± 1.5% at 1000 µg/mL. Enzyme inhibition studies showed significant inhibition of α-amylase (58.45 ± 0.7% to 87.20 ± 2.1%) and α-glucosidase (83.87 ± 1.6% at 1000 µg/mL) compared to acarbose. Glucose uptake in yeast cells increased to 59.87 ± 0.3% at 1000 µg/mL. In alloxan-induced diabetic mice, Cu NPs (150 mg/kg) reduced fasting blood glucose from 287 ± 4.2 to 131 ± 7.02 mg/dL over 30 days and improved lipid profiles (e.g., decreased total cholesterol to 85 ± 1.3 mg/dL and increased HDL to 39.8 ± 1.2 mg/dL). Histopathological analyses showed restoration of pancreatic and hepatic architecture. Collectively, chia seed polysaccharide-mediated copper-based nanoparticles exhibited anti-hyperglycemic, anti-inflammatory, and lipid-modulating activities under the investigated experimental conditions, supporting their further evaluation for diabetes-related applications.

## 1. Introduction

Diabetes mellitus is a chronic metabolic disorder characterized by persistent hyperglycemia resulting from insulin insufficiency, insulin resistance, or both [1,2]. It also disrupts carbohydrate, lipid, and protein metabolism, contributing to organ dysfunction, morbidity, and mortality [3,4]. Recent studies highlight diabetes as a systemic disease linked with oxidative stress, chronic inflammation, and metabolic dysregulation [5]. Chronic inflammation plays a crucial role in diabetes pathophysiology, with elevated pro-inflammatory mediators such as tumor necrosis factor-α, interleukin-6, and C-reactive protein frequently observed [6,7]. These mediators disrupt insulin signaling, worsening insulin resistance [8]. Furthermore, hyperglycemia promotes reactive oxygen species (ROS) generation, activating inflammatory signaling pathways, including nuclear factor-κB and mitogen-activated protein kinase pathways [9–11]. The interplay between oxidative stress and inflammation exacerbates metabolic disturbances and increases the risk of diabetes-related complications [12,13].

Traditional diabetic treatments, including biguanides, sulfonylureas, thiazolidinediones, and α-glucosidase inhibitors, focus on regulating blood glucose while preventing inflammation and oxidative stress [14]. However, common side effects like gastrointestinal discomfort, hypoglycemia, hepatotoxicity, weight gain, and an increased susceptibility to cardiovascular disease are linked to prolonged use [15]. These limitations underscore the need for novel therapeutic strategies that target both metabolic and inflammatory pathways [16].

Nanotechnology offers promising therapeutic potential due to the unique physicochemical properties of nanoparticles, including high surface-to-volume ratios and strong surface interactions [17,18]. Metal nanoparticles, especially copper, have shown promise in modulating antioxidant activity, enzyme activity, and inflammatory signaling pathways [19,20]. However, traditional synthesis methods often rely on toxic chemical reagents, limiting hemocompatibility and healthcare applications [21]. Eco-friendly synthesis approaches, such as using plant polysaccharides, offer a safer alternative by reducing and stabilizing nanoparticles naturally [22,23]. *Salvia hispanica* L. (chia) seeds release polysaccharide-rich mucilage when soaked in water and have shown several health benefits, including antibacterial, antihypertensive, antioxidant, anti-asthmatic, hypoglycaemic, and hypolipidaemic effects [24].

This study aims to synthesize copper-based nanoparticles via green synthesis using polysaccharides from *Salvia hispanica* seed mucilage. After evaluating their hemocompatibility, the nanoparticles’ in vitro anti-inflammatory and antidiabetic properties were assessed, followed by an in vivo evaluation using an alloxan-induced diabetic mouse model.

## 2. Materials and methods

### 2.1 Approval of the study

The Graduate Study Committee (GSC) of the Department of Biotechnology, Abdul Wali Khan University Mardan (AWKUM), Pakistan, recommended the study, and the Advanced Study and Research Board (ASRB), AWKUM, approved it. Ethical approval was obtained from the Institutional Animal Care and Ethics Committee (certificate # AWKUM/BIOTECH/2024/173).

### 2.2 Inclusivity in global research

This study was designed and conducted with substantial involvement of local researchers from Pakistan, where the experimental work and biological sample collection were performed. Chia seeds were obtained locally from Mardan, Khyber Pakhtunkhwa, Pakistan, and the in vitro and in vivo work was carried out under the supervision of the Department of Biotechnology at Abdul Wali Khan University Mardan, Pakistan. Human blood samples were collected from healthy volunteers after written informed consent, and animal experiments were performed after approval from the Institutional Animal Care and Ethics Committee of the same institution.

Local investigators contributed to the study design, experimental work, data curation and interpretation, manuscript preparation, and authorship. International collaborators contributed to data validation, formal analysis, interpretation, manuscript review, and related activities. The study did not involve indigenous populations, indigenous lands, cultural artifacts, human remains, or unauthorized export of biological materials. Additional ethical, cultural, and scientific details are provided in the completed PLOS Inclusivity in Global Research questionnaire submitted as Supporting Information.

### 2.3 Chemicals & reagents

All chemicals and reagents used in this study were of analytical reagent (AR) grade and were obtained from certified commercial suppliers without further purification. The copper(II) precursor (CuSO_4_·5H_2_O) used for nanoparticle synthesis was of analytical grade. The principal reagents used for the biochemical and biological assays included ethanol, phosphate-buffered saline (PBS), sodium carbonate, 3,5-dinitrosalicylic acid (DNS), *p*-nitrophenyl-α-D-glucopyranoside (pNPG), α-amylase, α-glucosidase, soluble starch, and Triton X-100. All experimental solutions were prepared using ultrapure distilled water. Glassware used for polysaccharide extraction, nanoparticle synthesis, and biological assays was thoroughly cleaned, rinsed with distilled water, and dried before use to minimize experimental contamination. Experiments were conducted under appropriate laboratory conditions as required for the respective procedures.

### 2.4 Collection of Seeds

Chia (*Salvia hispanica* L.) seeds were obtained from a local market in Mardan district, Khyber Pakhtunkhwa, Pakistan. The seeds were cleaned, sealed in sterile polyethene bags, and stored in a dry environment until further processing.

### 2.5 Preparation of water-soluble polysaccharides

Chia water-soluble polysaccharides were obtained following a modified aqueous extraction and ethanol precipitation method described earlier [25]. Briefly, 20 g of chia seeds were soaked in 1 L of distilled water and continuously stirred on a magnetic stirrer at ambient temperature for 24 h to release mucilage. After 24 h, the resulting mucilage was separated by filtration through a muslin cloth. The polysaccharides were precipitated with absolute ethanol under sterile conditions and dried in a rotary evaporator at 45 °C. The dehydrated polysaccharides were powdered and kept at −20 ℃ until further use.

### 2.6 Green synthesis of copper-based nanoparticles (Cu NPs)

Biosynthesis of Cu NPs was performed using chia seed polysaccharides as a green reducing and stabilizing agent [26]. Briefly, 0.1 g of chia seed polysaccharides was dissolved in 50 mL of distilled water to prepare the polysaccharide solution, which was then heated at 60 °C under constant stirring. Subsequently, 50 mL of 0.1 M copper(II) sulfate pentahydrate (CuSO_4_·5H_2_O) solution was added dropwise to the polysaccharide solution under continuous stirring. The reaction mixture was maintained at 60 °C with constant stirring for 4 h. The formation of Cu NPs was initially indicated by a visible color change from light blue to dark brown. After the reaction was completed, the mixture was cooled to room temperature and dried in an oven at 80 °C to obtain powdered Cu NPs.

### 2.7 Characterization of green-synthesized Cu NPs

#### 2.7.1. Visible observation.

Chia seed polysaccharides were used as the reducing agent in the green synthesis of Cu NPs. The formation of Cu NPs was evident from the dark brown precipitate.

#### 2.7.2. Ultraviolet-visible spectroscopy.

An ultraviolet-visible spectrometer (UV-1800/ Kyoto, Japan) was used to measure the Surface Plasmon Resonance (SPR) of Cu NPs. The highest absorption was measured by scanning the spectrum between 200 and 900 nm.

#### 2.7.3. Fourier transform infrared (FTIR) spectroscopy.

FTIR analysis was performed to identify the functional groups involved in Cu ion reduction and nanoparticle stabilization. The FTIR spectra were measured over the 4000–500 cm^−1^ range.

#### 2.7.4. X-ray diffraction (XRD) analysis.

The crystalline characteristics of the synthesized Cu NPs were observed via X-ray diffraction analysis. Diffraction patterns were recorded through an X-ray diffractometer (Bruker, D8 Advance, Germany) over a 2θ-scanning range of 20–80°.

The average crystallite size was estimated from the XRD diffraction data using the Debye–Scherrer equation:


$$\begin{array}{c} D=K\lambda /(\beta \text{cos }\theta ), \end{array}$$

where Dis the crystallite size, Kis the shape factor (0.9), λis the wavelength of the X-ray radiation, βis the full width at half maximum (FWHM) of the selected diffraction peak expressed in radians, and θis the corresponding Bragg angle.

#### 2.7.5. Scanning electron microscopy (SEM) analysis.

The surface morphology and morphological features of Cu NPs were examined through a scanning electron microscope (Hitachi S-4300SE, Tokyo, Japan) at various magnifications.

### 2.8 Hemocompatibility assessment

The hemocompatibility of green-synthesized Cu NPs was assessed using a human red blood cell (HRBC) hemolysis assay with slight modifications to the procedure described elsewhere [27]. Healthy human blood was collected from healthy volunteers with informed written consent on January 15, 2025, and diluted with phosphate-buffered saline (PBS, pH 7.4). The erythrocytes were centrifuged at 3000 rpm for 10 minutes and washed several times with PBS until the supernatant was clear. The erythrocytes were mixed with Cu NPs (250−1000 µg/mL) and incubated at 37 ℃. The absorbance of the supernatant was measured at 570 nm. Negative and positive controls were distilled water and 0.5% Triton X-100, respectively.


$$\begin{array}{c} \%\;Cytotoxic\;effect\;=\frac{\;Absorbance\;of\;test\;sample-Absorbance\;of\;Blank}{Absorbance\;of\;Triton-Absorbance\;of\;Control}*\text{1}\text{0}\text{0} \end{array}$$

### 2.9 In Vitro anti-inflammatory activities

#### 2.9.1 Human red blood cell (HRBC) membrane stabilization assay.

The anti-inflammatory potential of Cu NPs was evaluated using the human red blood cell (HRBC) membrane stabilization assay, as previously described [28]. Blood samples from healthy volunteers were mixed with Alsever’s solution, centrifuged, and washed with isotonic saline until a clear pellet was obtained. To preserve the erythrocyte pellet, it was resuspended in phosphate-buffered saline (PBS) at a 1:3 ratio and incubated with Cu NPs (250–1000 µg/mL) for 30 min at ambient temperature. The supernatant was transferred to a 96-well microplate, and HRBC hemolysis was measured at 560 nm. Diclofenac sodium was used as the reference drug, and distilled water as the negative control.


$$\begin{array}{c} \%\;HRBCs\;Membrane\;Stabilization\;=\frac{\;OD\;of\;test\;sample-OD\;of\;Blank}{OD\;of\;Triton-OD\;of\;Control}*\text{1}\text{0}\text{0} \end{array}$$

#### 2.9.2 Heat-induced hemolysis assay.

The effect of Cu NPs on heat-induced hemolysis was determined using the methodology reported elsewhere [29]. Blood was collected from a healthy, non-smoking donor. Alsever’s solution was gently mixed with 0.02 mL of whole blood from a healthy, non-smoking donor. The mixture was treated with Cu NPs (250–1000 µg/mL) and incubated at 56 ℃ for 30 min. Normal saline (20 mL) was added to prevent unwanted hemolysis. The resulting suspension was centrifuged at 3000 rpm for 10 min to separate phases. The absorbance of the supernatant was measured at 540 nm. To determine the hemolytic baseline, the blood was diluted 100-fold with distilled water. Diclofenac sodium was used as the positive control. The following equation was used to calculate the percent hemolysis:


$$\begin{array}{c} \%\;Heat\;Hemolysis\;=\frac{\;OD\;of\;test\;sample-OD\;of\;Blank}{OD\;of\;Triton-OD\;of\;Control}*\text{1}\text{0}\text{0} \end{array}$$

### 2.10 In Vitro antidiabetic potential

#### 2.10.1 α-amylase inhibition assay.

The 3,5-dinitrosalicylic acid (DNS) assay was used to determine the α-amylase inhibitory effect of Cu NPs [30]. The reaction mixture consisted of porcine pancreatic α-amylase (500 µL) prepared in PBS, Cu NPs at different concentrations, and a standard drug, and was incubated at 37 °C for 20 min. Thereafter, PBS (100 mM) and a 1% (w/v) starch solution were added, and the mixture was incubated again at the same temperature for 60 min. The enzyme reaction was stopped by adding dinitrosalicylic acid (DNS) reagent and heating for 10 minutes. The mixture was cooled, and absorbance was measured at 540 nm.

#### 2.10.2 α-glucosidase inhibition assay.

The α-glucosidase inhibitory potential of Cu NPs was evaluated according to the protocol reported previously [8]. Briefly, α-glucosidase (0.075 U) was incubated with Cu NPs at various concentrations (250–1000 µg/mL) at 37 ℃. The enzymatic reaction was initiated by adding 3 mM *p*-nitrophenyl-α-D-glucopyranoside (*p*-NPG) as a substrate, and the reaction was incubated for 20 min at a constant temperature. The reaction was stopped by adding sodium carbonate (Na_2_CO_3_). The release of *p*-nitrophenol was spectrophotometrically quantified at 540 nm. The percent inhibition of α-glucosidase and α-amylase was estimated using the following equation:


$$\begin{array}{c} \%\;Enzyme\;inhibition=\frac{\;OD\;of\;Test\;Sample-OD\;of\;Blank}{OD\;of\;Control}*\text{1}\text{0}\text{0} \end{array}$$

#### 2.10.3 Glucose uptake by yeast cells.

Glucose uptake by yeast cells was assessed using a previously described protocol [31]. Cu NPs (250, 500, and 1000 µg/mL) were mixed with 0.1 mL of 5 mM glucose solution and incubated at 37 °C for 10 minutes. The reaction was initiated by adding a 10% (v/v) yeast cell suspension, then vortexing and incubating at 37 °C for 40 min. The mixture was centrifuged at 1500 rpm for 10 min, and the residual glucose in the supernatant was spectrophotometrically estimated at 520 nm using the following formula:


$$\begin{array}{c} \%\;Yeast\;Glucose\;Uptake=\frac{\;OD\;of\;Test\;Sample-OD\;of\;Blank}{OD\;of\;Control}*\text{1}\text{0}\text{0} \end{array}$$

### 2.11 In Vivo antidiabetic studies

#### 2.11.1 Experimental animals and housing conditions.

Adult albino mice, 4 weeks old and weighing 24–29 g, were obtained from the Veterinary Research Institute, Peshawar, Pakistan. All animal procedures were approved by the Institutional Animal Care and Ethics Committee of the Department of Biotechnology, Abdul Wali Khan University Mardan, Pakistan, and were conducted in accordance with institutional and internationally accepted guidelines for laboratory animal care and use. A total of 30 mice (male and female) were used, with 5 animals per group. Mice were acclimatized for 7 days before the experiment and housed in clean cages with standard bedding under controlled conditions (22 ± 2 °C, 60 ± 5% relative humidity, and a 12:12 h light-dark cycle, with free access to standard laboratory chow and water).

The experimental duration was 30 days after treatment initiation. Mice were randomly allocated into six groups: Group I, normal control; Group II, diabetic control; Group III, diabetic mice treated with Cu NPs at 50 mg/kg body weight; Group IV, diabetic mice treated with Cu NPs at 100 mg/kg body weight; Group V, diabetic mice treated with Cu NPs at 150 mg/kg body weight; and Group VI, diabetic mice treated with metformin at 100 mg/kg body weight as the standard drug. Diabetes was induced in the designated groups as described in Section 2.11.2.

Animal health and welfare were monitored twice daily during the first 72 h after alloxan administration and at least once daily thereafter until the end of the experiment. We also observed during fasting, after alloxan injection, and after treatment administration. Monitoring included body weight, food and water intake, grooming, posture, locomotor activity, respiration, response to handling, hydration status, and signs of pain, distress, abnormal behavior, hypoglycemia, hyperglycemia, or moribund condition.

Humane endpoints were defined before the experiment. Any animal showing body-weight loss greater than 20% from baseline, severe or persistent lethargy, inability to access food or water, severe dehydration, labored breathing, persistent recumbency, convulsions, severe hypoglycemia, uncontrolled hyperglycemia with clinical deterioration, injection-site complications, unrelieved pain or distress, or moribund condition was to be removed from the study and humanely euthanized immediately. Death was not used as an experimental endpoint, and animals were not allowed to die without intervention. If a humane endpoint was reached, euthanasia was to be performed under deep anesthesia using ketamine, followed by cervical dislocation. Carcasses and biological waste were disposed of in accordance with institutional biosafety and animal waste disposal guidelines.

#### 2.11.2 Induction of diabetes.

After overnight fasting, diabetes was induced in the designated groups via an intraperitoneal injection of alloxan (170 mg/kg BW). The normal control group received the same volume of normal saline. Glucose levels were measured 48 h after alloxan administration, and animals with fasting sugar levels exceeding 140 mg/dL were considered diabetic.

#### 2.11.3 Blood glucose monitoring.

Whole blood was collected from the tail vein after 12–16 h of fasting. A glucometer was used to measure fasting glucose levels on days 1, 5, 10, 15, 20, 25, and 30.

#### 2.11.4 Histopathological examination.

Pancreatic and hepatic tissues were removed, washed with phosphate-buffered saline, and stored in 15% formaldehyde solution. The samples underwent standard histological processing. The tissues were sectioned, counterstained with eosin and hematoxylin, and observed under a light microscope.

#### 2.11.5 Blood biochemical parameters.

The animals were euthanized on the 30^th^ day of the initiation of treatment, and blood was collected through cardiac puncture. Serum was collected to determine triglyceride (TG), high-density lipoprotein (HDL), low-density lipoprotein (LDL), and total cholesterol (TC) levels using diagnostic kits.

### 2.12 In Vivo clinical toxicity assessment

Potential clinical signs of toxicity associated with copper-based nanoparticle administration were monitored at 100 and 150 mg/kg body weight for one month. During the trials, animals were monitored for behavioral changes, mortality, and body weight.

### 2.13 Statistical analysis

All in vitro experiments were repeated three times, and results are presented as mean ± standard deviation (SD). Two-way analysis of variance (ANOVA) was used to analyze in vitro assays with different treatments and multiple concentrations, followed by Tukey's multiple-comparison test. One-way analysis of variance (ANOVA), followed by Tukey's multiple-comparison test, was used for single-time-point in vivo parameters, including hematological and serum lipid indices. A two-way repeated-measures ANOVA with treatment group and time as factors, followed by Tukey’s multiple-comparison test, was used to analyze longitudinal blood glucose and body-weight measures. The Shapiro–Wilk and Levene's tests were used to assess normality and homogeneity of variance, respectively. A p-value of < 0.05 was considered statistically significant. In vitro assays were performed in triplicate (n = 3), and data are presented as mean ± SD. For the in vivo study, each animal was considered an independent biological replicate, with five animals per experimental group (n = 5).

## 3. Results

### 3.1 Green synthesis of Cu NPs using chia seed polysaccharides

The main indicator of the green-synthesized Cu NPs was a characteristic colorimetric change in the reaction mixture. Initially, the copper salt solution was blue; after adding chia seed polysaccharides, it turned dark brown (Fig 1).

**Fig 1:**
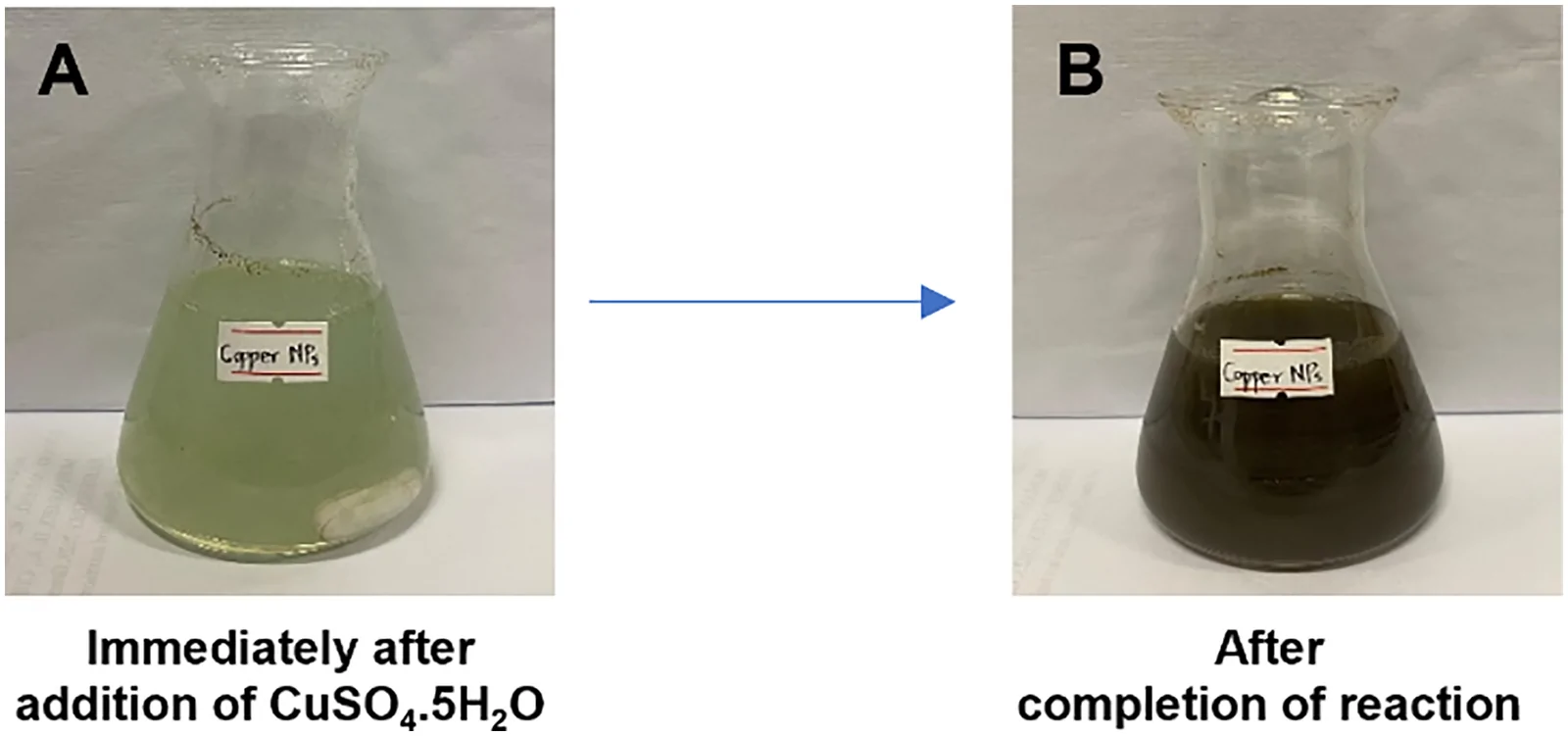
Optical monitoring of Cu NPs synthesis using the chia seed polysaccharide. (A) The reaction mixture was light blue immediately after adding CuSO_4_.5H_2_O. (B) After completion, the reaction mixture turned dark brown, indicating Cu NPs formation.

### 3.2 UV-Visible spectroscopic characterization of Cu NPs

To confirm Cu NPs synthesis and to determine their optical properties, UV-visible spectroscopy was used. The green chia seed polysaccharide-mediated Cu NPs exhibited a characteristic absorption band at 430 nm, indicative of surface plasmon resonance (Fig 2).

**Fig 2:**
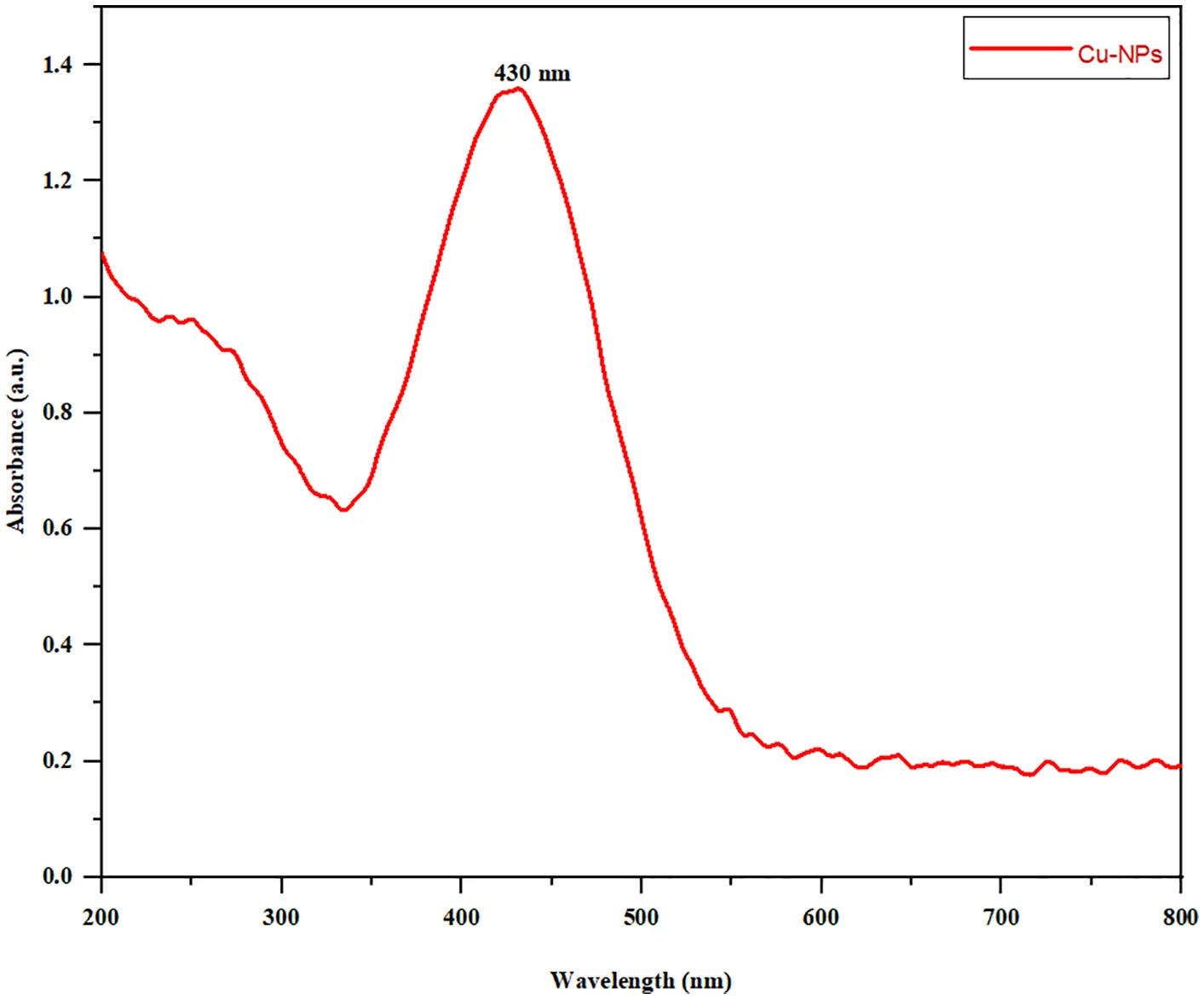
UV-VIS spectroscopic Analysis of Cu NPs.

### 3.3 Fourier transform infrared spectrophotometry (FTIR) of Cu NPs

FTIR spectroscopy was performed to investigate the functional groups involved in the reduction and stability of Cu NPs. The FTIR spectrum showed strong absorption peaks for the hydroxyl (–OH), carbonyl (C = O), and ether (C–O–C) functional groups (Fig 3). The presence of the O-H stretching vibration was confirmed by bands in the 3200–3400 cm^−1^ range, indicating the presence of polysaccharide hydroxyl groups in nanoparticle stabilization. Peaks observed between 1600 and 1650 cm^−1^ were attributed to the C = O stretching vibrational band, while bands around 1000–1100 cm^−1^ were assigned to the C-O stretching vibrational band. The FTIR findings are consistent with the involvement of chia seed polysaccharide functional groups in the synthesis and stabilization of the copper-based nanoparticle formulation.

**Fig 3:**
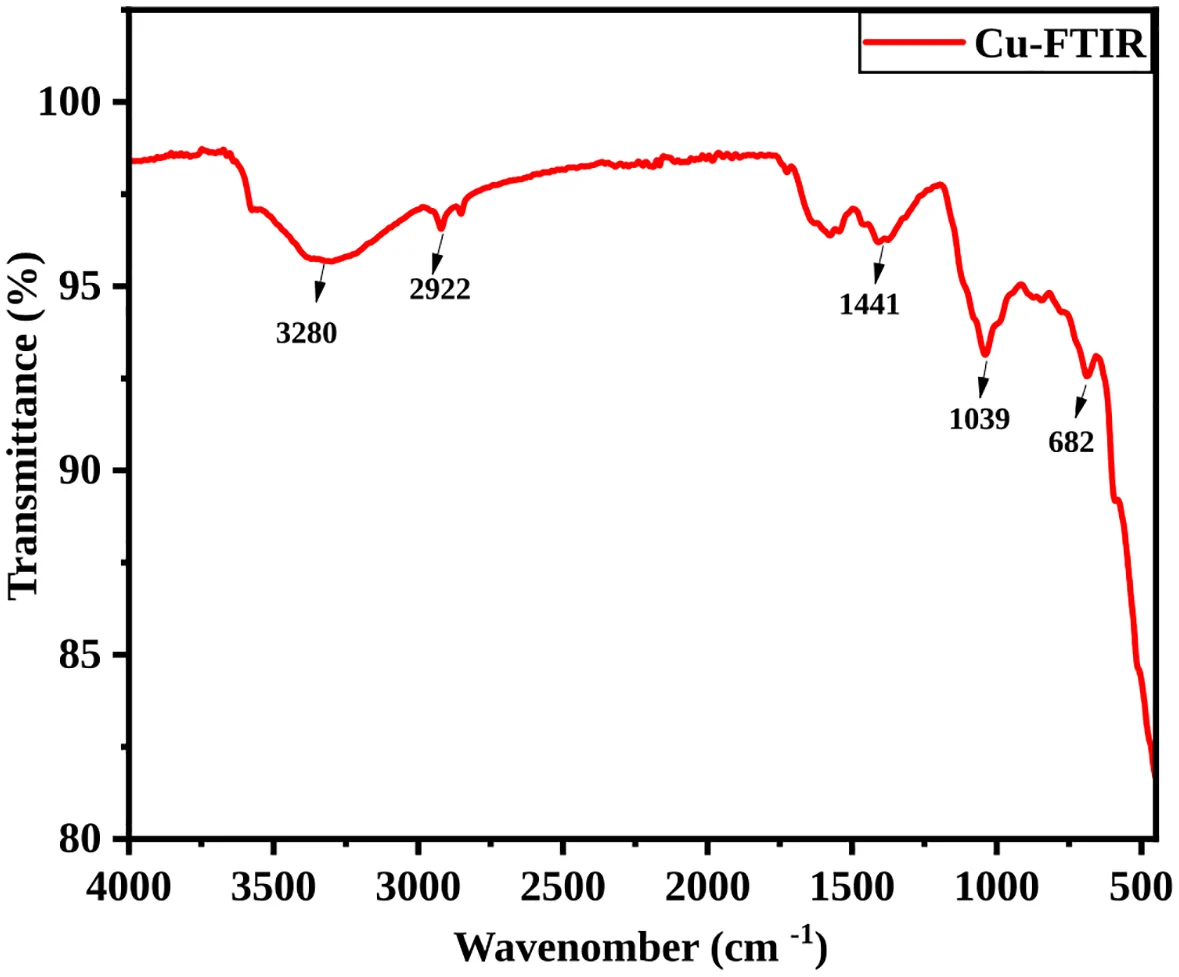
FTIR analysis of Cu NPs.

### 3.4 X-Ray diffraction (XRD) of Cu NPs

The crystal structure of the synthesized Cu NPs was analyzed using X-ray diffraction. The XRD pattern shows distinct diffraction reflections (Fig 4), indicating that the synthesized nanomaterial is crystalline. Selected characteristic diffraction peaks around 27.86°, 32.26°, 38.16°, 46.18°, and 57.52° (2θ) were used for Debye–Scherrer analysis. The calculated crystallite sizes for these reflections were 26.02 nm, 18.26 nm, 21.93 nm, 20.22 nm, and 38.41 nm, respectively, yielding a mean crystallite size of 24.97 nm. These values represent the dimensions of coherently diffracting crystalline domains, and not necessarily the physical particle size as seen by SEM, because a single particle can contain one or more crystallites. Some additional reflections were also evident in the diffraction pattern; however, XRD alone could not conclusively establish their origin.

**Fig 4:**
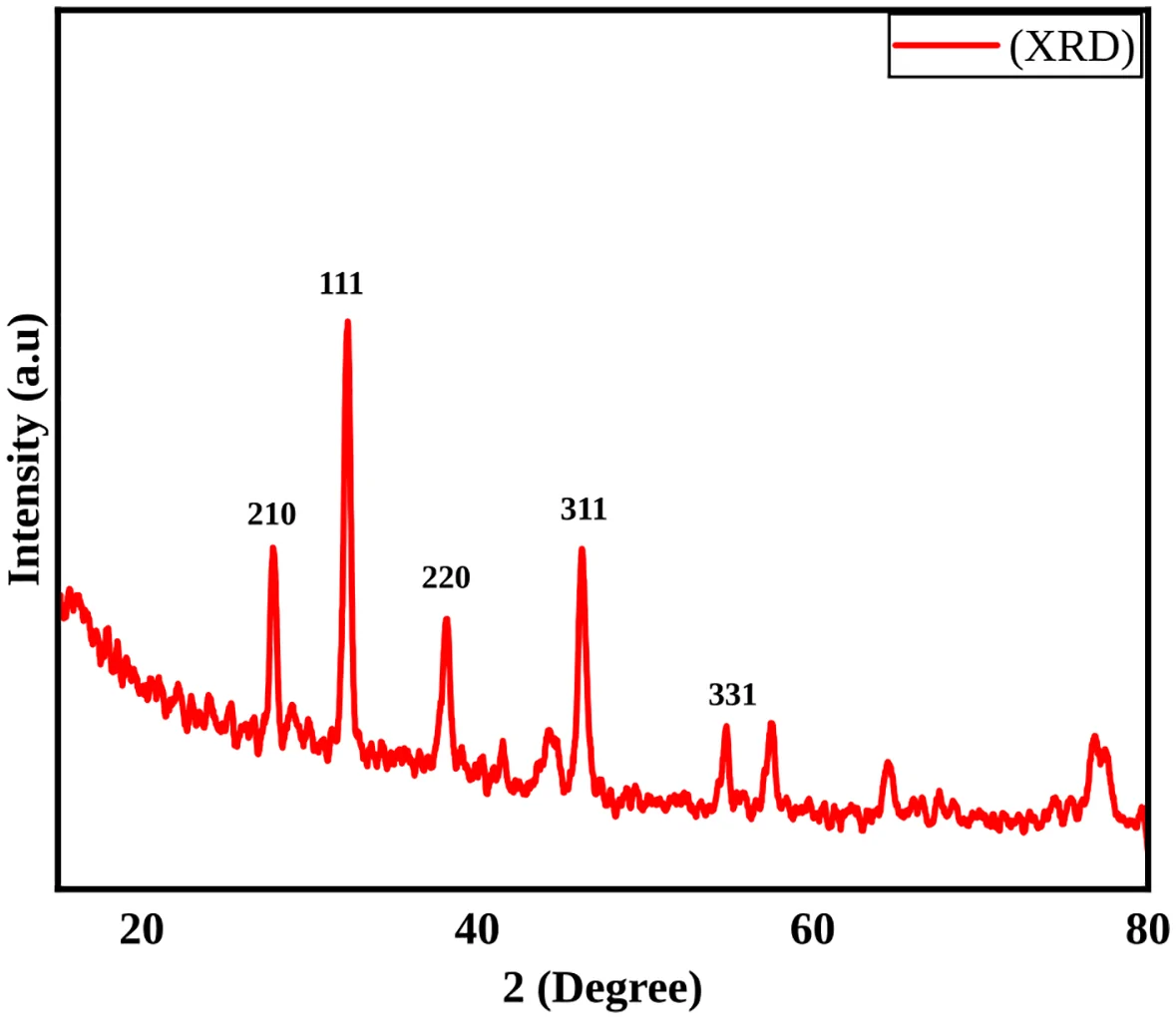
X-ray diffraction peaks of Cu NPs.

### 3.5 Morphological analysis of Cu NPs by scanning electron microscope (SEM)

To investigate the surface morphology of the synthesized Cu NPs, a scanning electron microscope (Hitachi S-4300SE, Tokyo, Japan) was used. SEM images of Cu NPs indicated that the synthesized nanoparticles were evenly dispersed and exhibited a spherical morphology at the nanoscale (Fig 5). A certain degree of agglomeration was observed, which might be due to polysaccharide constituents. Agglomeration resulted in irregularly shaped nanoparticles, likely due to physical bonding and influenced by the solvent used.

**Fig 5:**
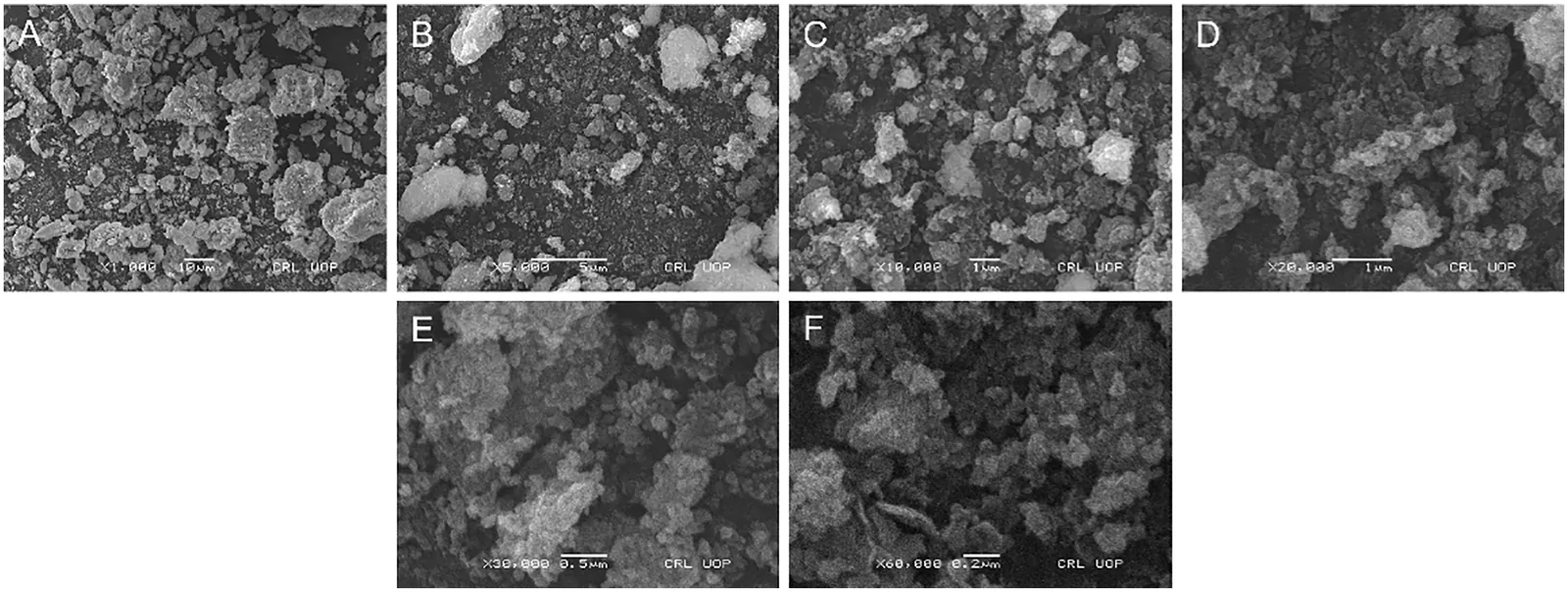
SEM images of chia seed polysaccharide-mediated Cu NPs at different magnifications: (A) ×1,000, (B) ×2,700, (C) ×3,000, (D) ×5,500, (E) ×9,500, and (F) ×10,000.

### 3.6 Hemocompatibility of Cu NPs

The hemolytic activity of chia polysaccharides and Cu NPs was evaluated at 250–1000 µg/mL using Triton X-100 as the positive control. Chia seed polysaccharides exhibited negligible hemolysis across all the tested concentrations, with a percent hemolysis of 0.069 ± 0.04% at 250 µg/mL, 1.18 ± 0.09% at 500 µg/mL, and 1.72 ± 0.13% at 1000 µg/mL (Fig 6). Cu NPs exhibited a dose-responsive increase in hemolytic activity, showing 0.92 ± 0.06% at 250 µg/mL, 5.71 ± 0.44% at 500 µg/mL, and 8.42 ± 0.63% at 1000 µg/mL. Triton X-100 induced 99.49 ± 1.12% hemolysis, confirming complete erythrocyte lysis. These findings demonstrate concentration-dependent hemocompatibility of the Cu NPs, with increasing hemolytic activity at higher nanoparticle concentrations.

**Fig 6:**
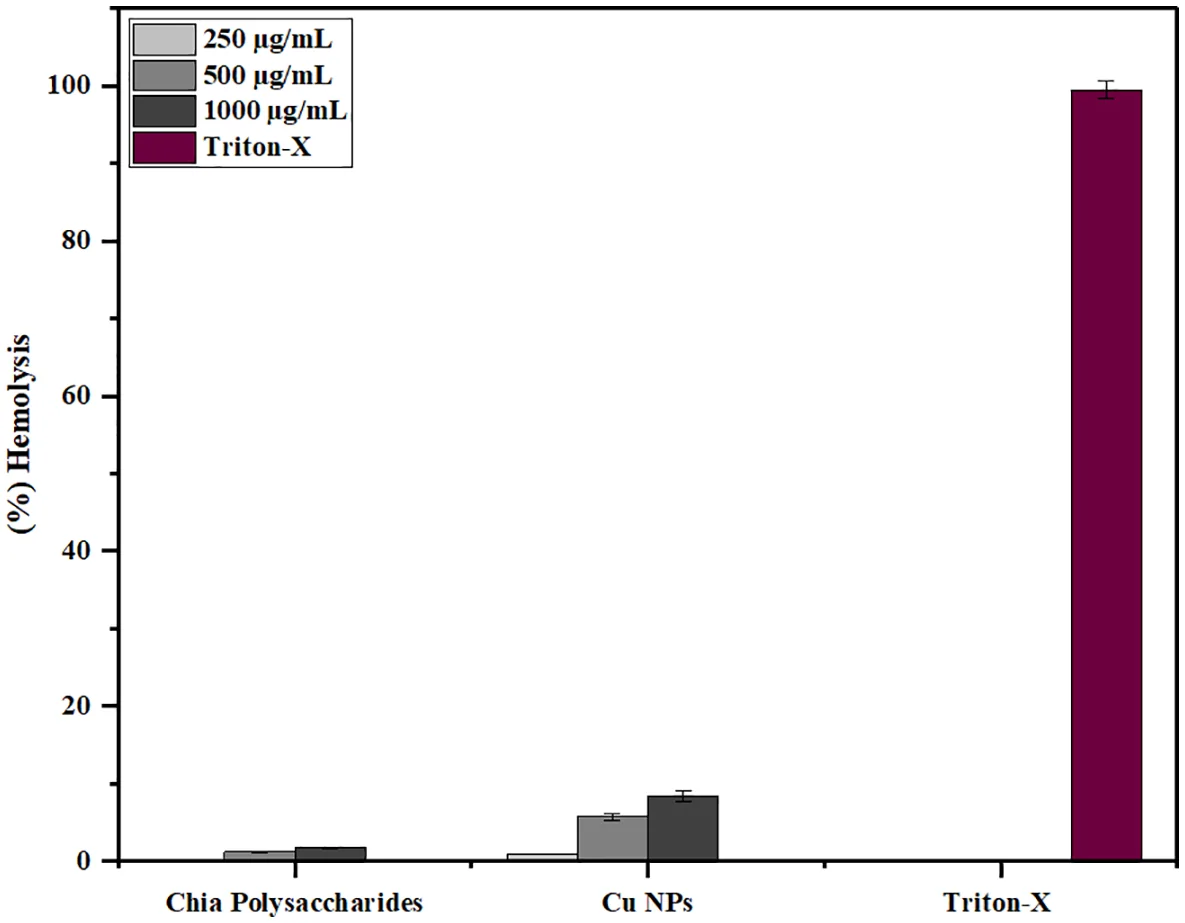
Hemolytic Activity of chia seed polysaccharides and Cu NPs evaluated at different concentrations, indicating their concentration-dependent hemocompatibility towards erythrocytes.

### 3.7 In Vitro anti-inflammatory activities of Cu NPs

#### 3.7.1 Membrane stabilization activity of Cu NPs in human red blood cells.

Membrane stabilization activity of chia polysaccharide-mediated Cu NPs was evaluated using human red blood cells (HRBCs). The Cu NPs exhibited a dose-dependent inhibitory effect on hemolysis (Fig 7A). At 250, 500, and 1000 µg/mL, the Cu NPs inhibited hemolysis by 45.83 ± 1.7%, 57.59 ± 1.4%, and 70.59 ± 2.1%, respectively, compared to 95.48 ± 0.92% inhibition by diclofenac sodium at 1000 µg/mL. The results were statistically significant (p-value < 0.0005).

**Fig 7:**
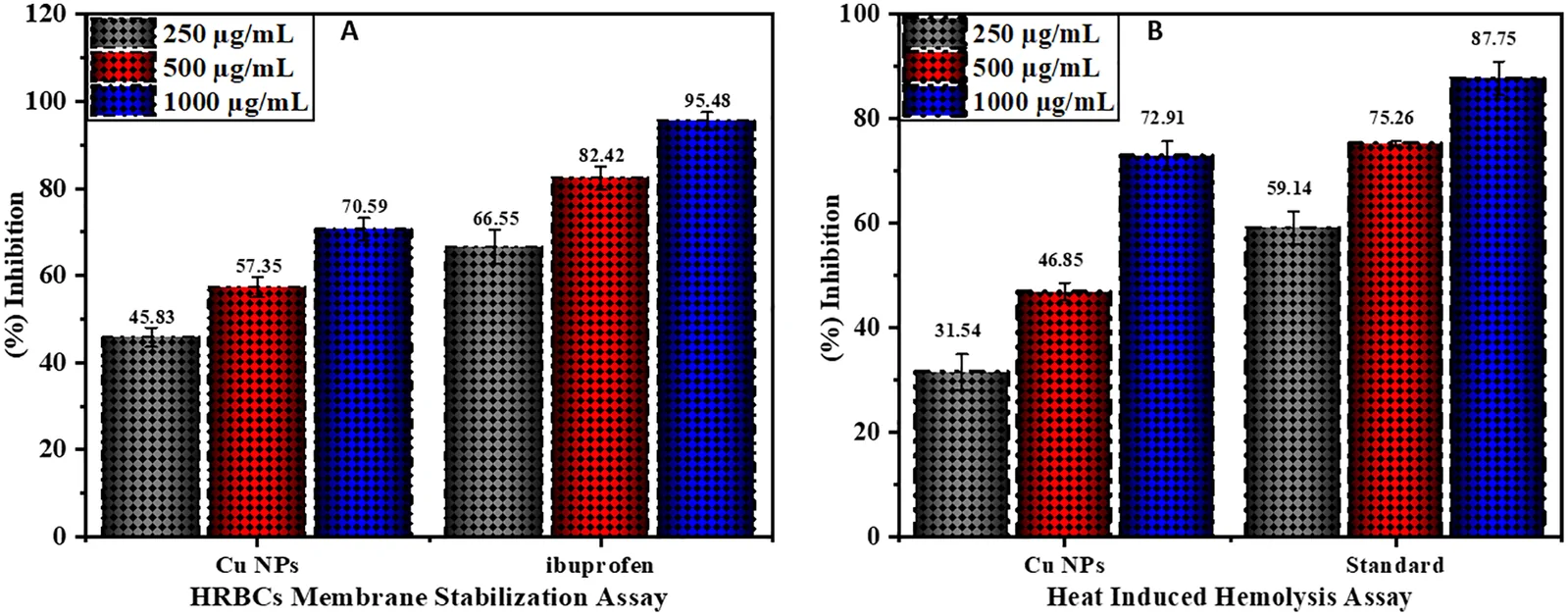
Effects of Cu NPs and the standard drug on (A) Human red blood cell (HRBC) membrane stabilization and (B) heat-induced hemolysis, demonstrating their anti-inflammatory potential. Data are presented as mean ± SD of three replicate measurements (n = 3). Error bars represent SD.

#### 3.7.2 Heat-induced hemolysis inhibition by Cu NPs.

A heat-induced hemolysis assay was used to assess the in vitro anti-inflammatory activity of Cu NPs at selected concentrations. The effect of green-synthesized Cu NPs was comparable to that of diclofenac sodium (Fig 7B). The data showed that diclofenac sodium exhibited the highest anti-inflammatory effect (87.75 ± 1.8%) at 1000 µg/mL, whereas Cu NPs showed 72.91 ± 1.5% inhibition at the same concentration. At the lowest tested concentration (250 µg/mL), diclofenac sodium exhibited an inhibition of 59.14 ± 1.9%, while Cu NPs showed 31.54 ± 2.6% inhibition.

### 3.8 In Vitro antidiabetic activities of Cu NPs

The in vitro antidiabetic potential of green-synthesized Cu NPs was assessed using α-amylase and α-glucosidase inhibition assays and a yeast glucose uptake assay.

#### 3.8.1 α-Amylase inhibition activity of Cu NPs.

The α-amylase inhibitory activity of green-synthesized Cu NPs was evaluated at 250–1000 µg/mL and compared with acarbose as the reference inhibitor. Cu NPs exhibited concentration-dependent inhibition of 58.45 ± 0.7%, 74.11 ± 2.5%, and 87.20 ± 2.1% at 250, 500, and 1000 µg/mL, respectively, whereas acarbose showed inhibition of 75.61 ± 2.3%, 87.74 ± 1.6%, and 99.21 ± 1.7% at the corresponding concentrations (Fig 8A). Since both Cu NPs and acarbose produced greater than 50% inhibition at the lowest tested concentration (250 µg/mL), their IC₅₀ values were below 250 µg/mL. A precise IC₅₀ could not be determined within the experimentally tested concentration range because concentrations below 250 µg/mL were not evaluated.

**Fig 8:**
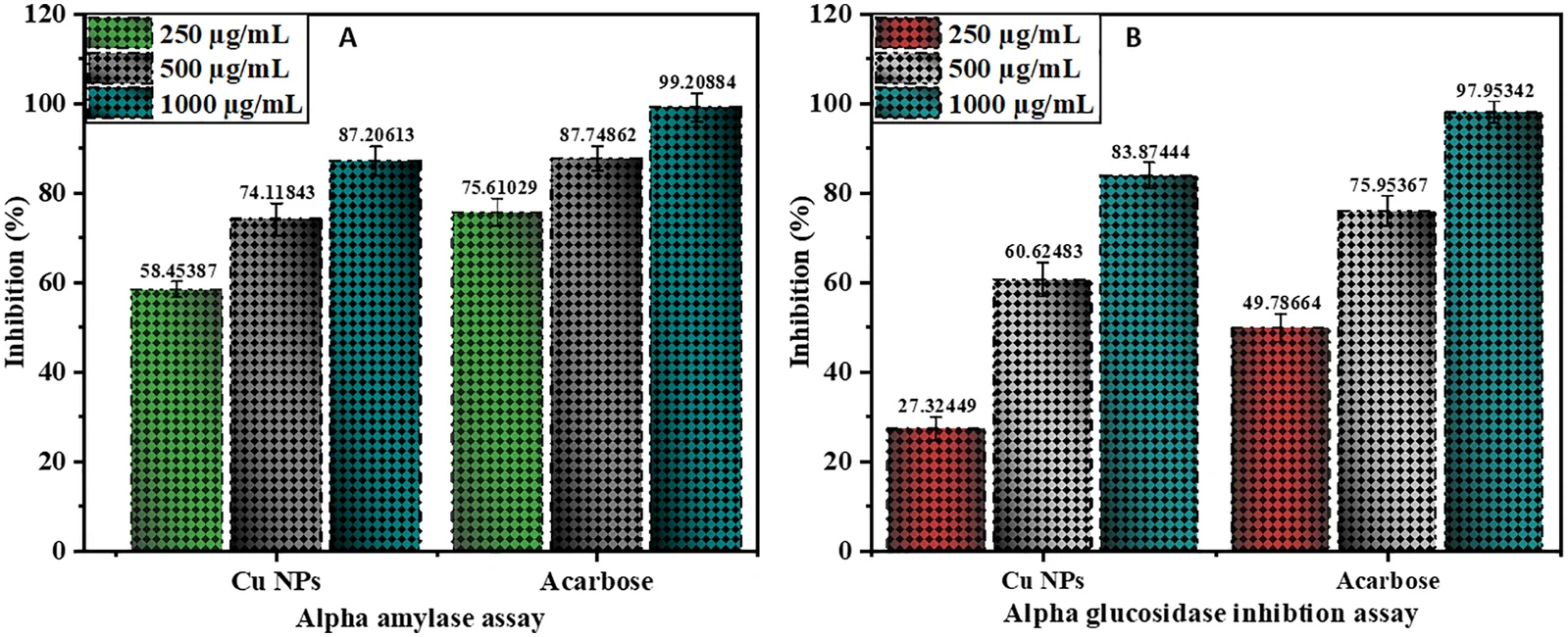
In vitro evaluation of antidiabetic potential of chia seed polysaccharide-mediated Cu NPs relative to acarbose via (A) α-amylase and (B) α-glucosidase inhibitory activities. Data are presented as mean ± SD of three replicate measurements (n = 3). Error bars represent SD.

#### 3.8.2 α-glucosidase inhibition activity of Cu NPs.

α-Glucosidase is a key enzyme located on the mucosal brush border of the small intestine and is essential for the enzymatic breakdown of complex carbohydrates into monosaccharides. α-Glucosidase inhibition reduces glucose absorption, minimizing postprandial glycaemic excursions and aiding diabetes management. Cu NPs exhibited concentration-dependent α-glucosidase inhibition of 27.32%, 60.62%, and 83.87% at 250, 500, and 1000 µg/mL, respectively. Acarbose exhibited corresponding inhibition values of 49.79%, 75.95%, and 97.95%. Based on interpolation of the experimentally observed concentration-response data surrounding 50% inhibition, the estimated IC₅₀ values were approximately 420.23 µg/mL for Cu NPs and 252.04 µg/mL for acarbose. These findings demonstrate greater inhibitory potency of the reference inhibitor under the experimental conditions.

#### 3.8.3 Yeast glucose uptake by Cu NPs.

The antidiabetic effects of Cu NPs were evaluated using a yeast cell glucose uptake assay. Cu NPs dose-dependently enhanced glucose uptake by yeast cells. At 250, 500, and 1000 µg/mL, the average glucose uptake by yeast cells was 17.35 ± 1.5%, 31.27 ± 0.8%, and 59.87 ± 0.3%, respectively. In comparison, the standard drug acarbose demonstrated glucose uptake of 42.35 ± 1.4%, 79.15 ± 0.3%, and 97.25 ± 0.7% at the same concentrations (Fig 9). These findings suggest that Cu NPs possess significant antidiabetic potential.

**Fig 9:**
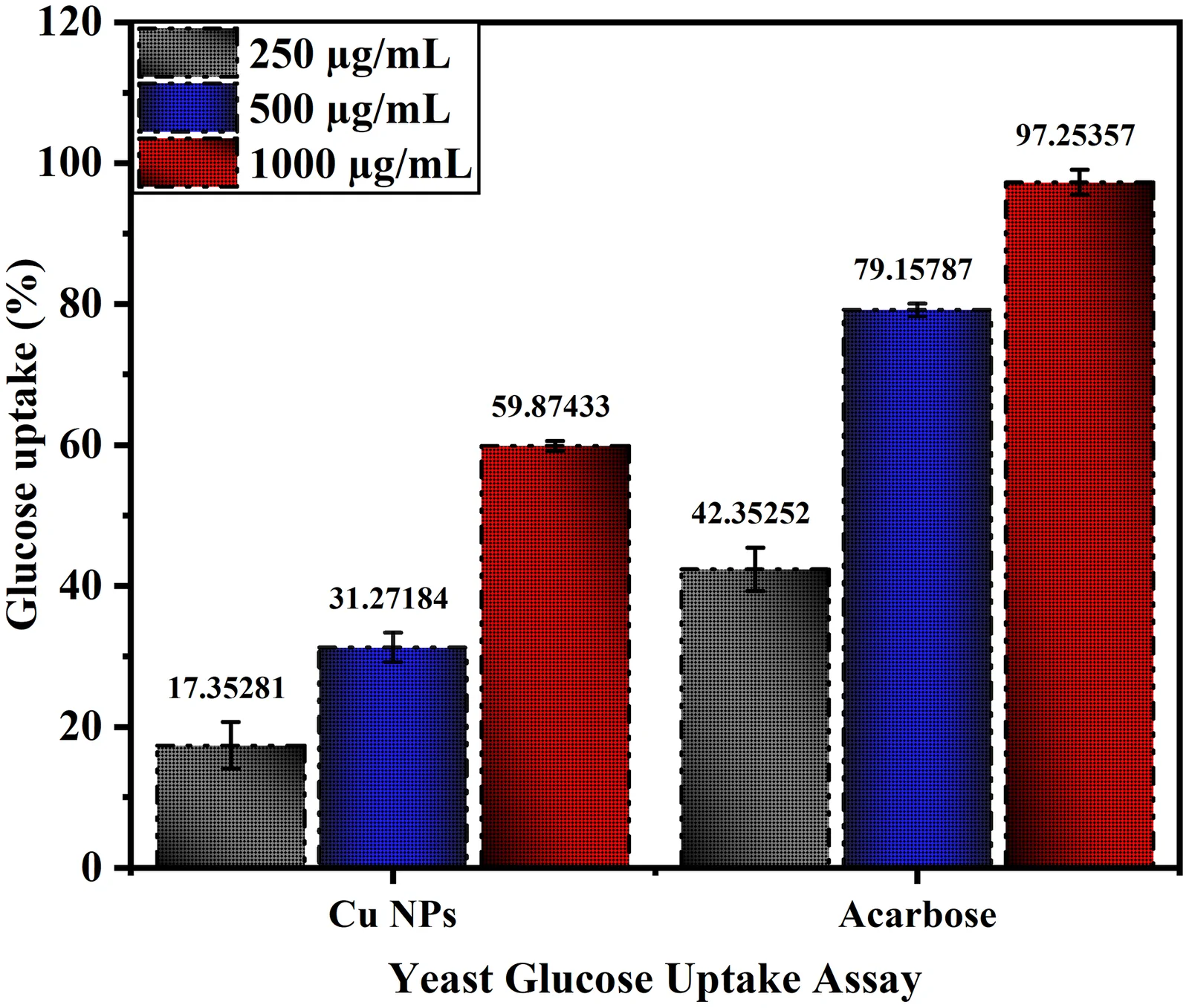
Effect of Cu NPs and standard drug on yeast glucose uptake activity, demonstrating their potential role in glucose utilization. Data are presented as mean ± SD of three replicate measurements (n = 3). Error bars represent SD.

### 3.9 In Vivo antidiabetic activity of Cu NPs in alloxan-induced diabetic mice

#### 3.9.1 Effect of Cu NPs on blood glucose levels.

Alloxan treatment elevated blood glucose levels in experimental mice. However, Cu NPs treatment of diabetic mice at 50, 100, and 150 mg/kg BW for 30 days significantly reversed alloxan-induced hyperglycemia (Fig 10). Blood glucose levels increased notably in the diabetic control group, increasing from 277 ± 5.1 to 420 ± 22.03 mg/dL. The normal control group showed no significant increase in blood glucose levels, which remained between 114 ± 2.7 and 119 ± 3.63 mg/dL throughout the experiment. Groups treated with Cu NPs showed a dose-dependent reduction in blood glucose levels. Mice treated with 50 mg/kg Cu NPs showed a decrease from 288 ± 5.0 to 160 ± 5.54 mg/dL, while those treated with 100 mg/kg Cu NPs exhibited a reduction from 290 ± 2.5 to 154 ± 5.033 mg/dL. Similarly, 150 mg/kg Cu NPs further reduced glucose levels from 287 ± 4.2 to 138 ± 7.50 mg/dL, and to 131 ± 7.024 mg/dL at the end of treatment. These findings demonstrate the anti-hyperglycemic effect of Cu NPs in alloxan-induced diabetic mice.

**Fig 10:**
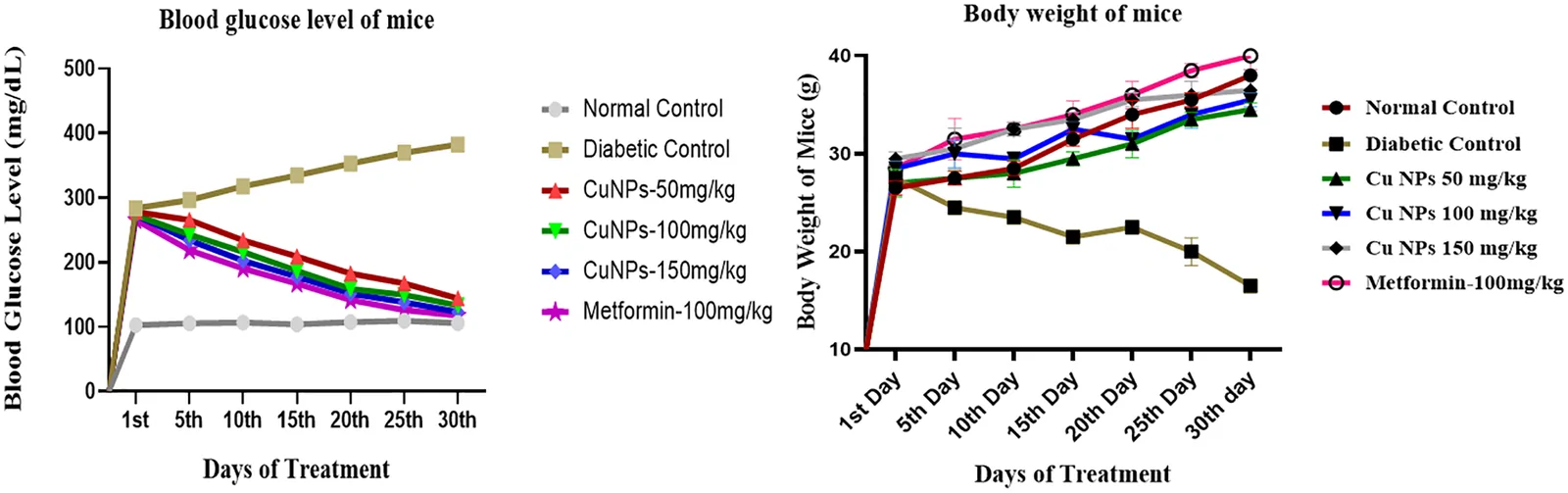
Effect of Cu NPs and the standard drug on the blood glucose levels and body weight of experimental mice. Data are presented as mean ± SD (n = 5 animals per group). Error bars represent SD.

#### 3.9.2 Effect of Cu NPs on hematological parameters.

Hematological findings showed a moderate increase in red blood cell (RBC) count in groups treated with varying doses of Cu NPs, accompanied by a significant reduction in white blood cell (WBC) count. The main hematological parameters, i.e., packed cell volume (PCV) and mean corpuscular volume (MCV), improved noticeably after treatment with a range of Cu NP doses. These outcomes demonstrate that Cu NP treatment positively modulates blood parameters within the experimental groups (Table 1).

**Table 1:** Hematological parameters of mice treated with Cu NPs. Values are presented as mean ± SD (n = 5 animals per group).

Groups	RBC (×10^6^ cells/μL)	WBC (×10^3^ cells/μL)	MCV fL (femtoliters)	PCV (%)
Normal Control	6.4 ± 1.7	5.6 ± 0.9	24.4 ± 3.1	47.1 ± 2.5
Diabetic Control	2.3 ± 2.7	2.8 ± 1.5	15 ± 1.6	25.5 ± 2.9
Cu NPs (50 mg/kg)	3.8 ± 2.1	3.9 ± 2.3	18.5 ± 1.3	33.8 ± 1.3
Cu NPs (100 mg/kg)	4.1 ± 3.5	4.7 ± 2.6	21.6 ± 2.7	38.1 ± 2.2
Cu NPs (150 mg/kg)	4.7 ± 2.7	5 ± 1.7	23 ± 3.1	41 ± 0.7
Standard (100 mg/kg)	5.5 ± 1.8	5.3 ± 0.8	23.8 ± 0.6	43.1 ± 1.3

#### 3.9.3 Effect of Cu NPs on serum lipid profiles.

Lipid profiles were evaluated in six groups of mice after 30 days of treatment. A significant improvement in lipid profile parameters was observed in Groups III-V treated with Cu NPs at different doses, as well as in Group VI, which received the standard drug metformin (100 mg/kg). Compared with the diabetic control group, these treated groups showed substantial reductions in total cholesterol, triglycerides, and LDL, along with a noticeable increase in HDL (Table 2).

**Table 2:** Effect of Cu NPs on lipid profile of experimental mice.

Groups	Total Cholesterol (mg/dL)	LDL (mg/ dL)	HDL (mg/ dL)	Triglycerides (mg/ dL)
Normal Control	78 ± 0.4	21 ± 1.1	43.7 ± 2.5	131.3 ± 3.7
Diabetic Control	147 ± 3.3	43 ± 2.1	17.2 ± 1.4	194.7 ± 1.9
Cu NPs (50 mg/kg)	108 ± 2.7	31.5 ± 0.9	29.8 ± 0.2	143.5 ± 2.8
Cu NPs (100 mg/kg)	91.3 ± 4.1	28.2 ± 1.5	36 ± 2.7	161.9 ± 4.1
Cu NPs (150 mg/kg)	85 ± 1.3	25.5 ± 1.7	39.8 ± 1.2	177.2 ± 1.5
Standard (100 mg/kg)	80 ± 1.9	24.5 ± 2.1	41.2 ± 1.6	183.5 ± 3.3

#### 3.9.4 Histopathological evaluation of the hepatic tissues.

Histological examination of the normal control group revealed a well-preserved intact hepatic structure (Fig 11A). In contrast, the diabetic control group exhibited hepatocellular necrosis and severe structural degeneration of liver tissue (Fig 11B). Liver sections obtained from mice treated with 50 mg/kg BW of Cu NPs showed variations in hepatocyte vacuole dimensions (Fig 11C). However, higher doses of Cu NPs (100 mg/kg BW and 150 mg/kg BW) significantly reduced hepatic deterioration and hepatocellular necrosis (Fig 11D and E). Furthermore, the diabetic group treated with the standard drug at 100 mg/kg BW showed a histological profile comparable to the normal control group, suggesting significant restoration of hepatic architecture (Fig 12F).

**Fig 11:**
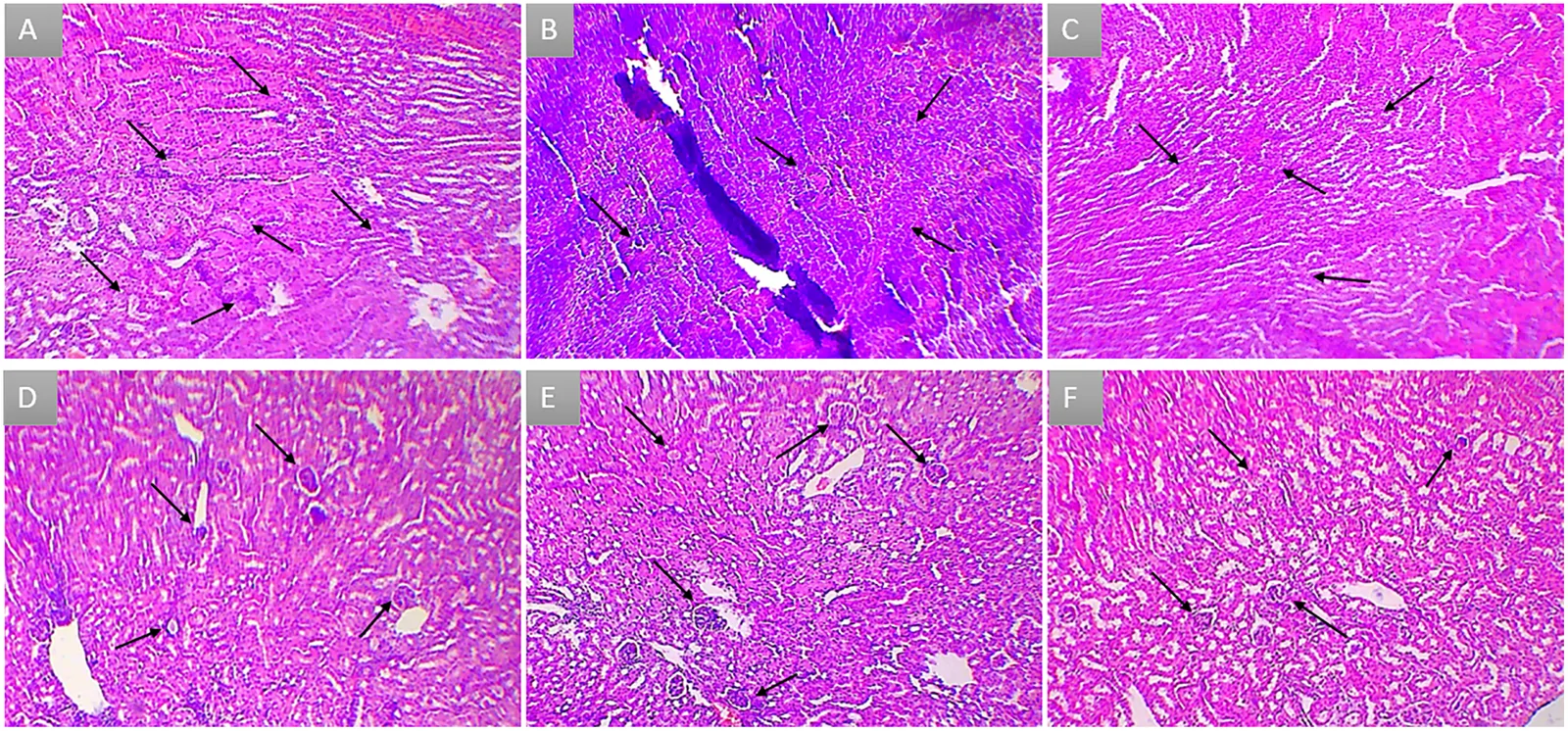
Histological micrographs of the hepatic tissue from different experimental groups. (**A**) Normal control showing intact hepatic structure with normal sinusoids and vacuoles. (**B**) Diabetic control demonstrating severe hepatocellular deterioration and necrosis. (**C-E**) Chia seed polysaccharide-mediated Cu NPs-treated group (50, 100, and 150 mg/kg BW), exhibiting progressive improvement with reduced necrosis and regeneration of hepatic sinusoids and vacuolar structures. (**F**) Standard-treated group (100 mg/kg BW) showing near-normal liver histology.

**Fig 12:**
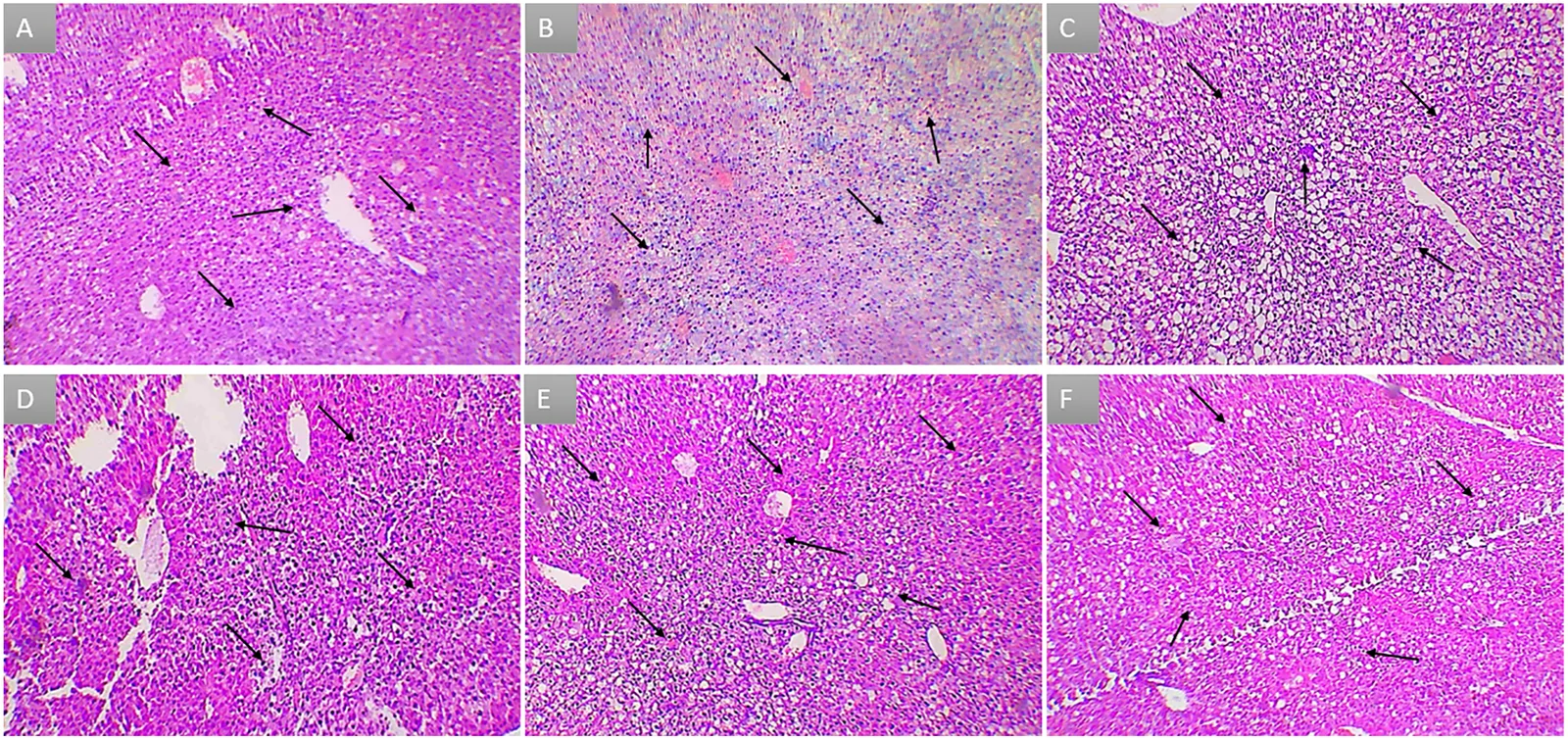
Histological micrographs of pancreatic tissue from different experimental groups. (**A**) Normal control showing intact lobular architecture and densely packed acinar cells. (**B**) Diabetic control showing marked β-cell destruction. (**C–E**) Chia seed polysaccharide-mediated Cu NP-treated groups at 50, 100, and 150 mg/kg BW, respectively, demonstrating dose-dependent restoration of pancreatic islets. (**F**) The standard-treated group (100 mg/kg BW) showed near-normal morphology of the islets of Langerhans.

#### 3.9.5 Histopathological evaluation of the pancreatic tissues.

Histological examination of the pancreas in the normal control group revealed a well-preserved and defined pancreatic structure (Fig 12A). The exocrine component was organized into small lobules densely populated with acinar cells, separated by septa of intact interlobular connective tissue. This pattern indicated normal pancreatic structural integrity (Fig 12A). In comparison, the diabetic control group showed noticeable pathological changes in both exocrine and endocrine components of the pancreas (Fig 12B). Various small vacuoles (indicated by an arrow) were observed within enlarged acinar cells. Severe β-cells damage was observed in the alloxan-induced diabetic mice. Pancreatic section from the diabetic group treated with 50 mg/kg BW of Cu NPs showed generalized architectural distortion, as shown in Fig 12C. Acinar injury was evident by cytoplasmic vacuolation and cellular atrophy in most exocrine acini. Treatment with 100 mg/kg BW of Cu NPs resulted in partial restoration of pancreatic islets, while medium-sized vacuoles were observed in the basal region of acinar cells (Fig 12D). Treatment with 100 mg/kg BW of Cu NPs resulted in partial restoration of pancreatic islets, while medium-sized vacuoles were observed in the basal region of acinar cells (Fig 12D). Treatment with 150 mg/kg BW of Cu NPs resulted in near-normal morphology of the islets of Langerhans (Fig 12E), while the standard-treated group showed restoration toward normal pancreatic islet architecture (Fig 12F).

### 3.10 In Vivo toxicity assessment of Cu NPs

During the one-month observation period, animals receiving the tested doses of Cu-based NPs showed no overt clinical signs of toxicity. No treatment-related mortality or obvious clinical abnormalities, including restlessness, respiratory distress, or coma, were observed. These findings are limited to the clinical observations and doses evaluated in the present study and should not be interpreted as evidence of general systemic safety.

## 4. Discussion

The increasing global incidence of diabetes mellitus has heightened the demand for therapeutic approaches capable of simultaneously modulating inflammation, hyperglycemia, and associated metabolic disorders [12,32,33]. In this context, green nanotechnology has attracted substantial attention as a viable platform for developing multifunctional antidiabetic agents [34]. In the current study, copper-based nanoparticles (Cu NPs) synthesized using chia (*Salvia hispanica*) seed polysaccharides showed significant anti-inflammatory and antidiabetic activities in vitro, supported by strong anti-hyperglycemic effects in alloxan-induced diabetic mice. These results align with and extend prior research on polysaccharide-mediated and green-synthesized copper-based nanoparticles, highlighting their biomedical significance [35–37].

Green synthesis using plant-derived polysaccharides offers distinct advantages over conventional chemical methods by eliminating toxic reducing agents and providing instant surface functionalization, thereby improving nanoparticle consistency and hemocompatibility [38,39]. Plant-derived polysaccharides are increasingly used in green synthesis because their abundant functional groups can reduce and stabilize metal nanoparticles [40]. Chia seed polysaccharides are rich in hydroxyl, carboxyl, and uronic acid moieties, which can reduce copper ions while stabilizing the resulting nanoparticles [41]. Comparable roles have been described for chitosan- and hyaluronate-capped CuO nanoparticles, where polysaccharide matrices efficiently control nucleation and growth while improving biological performance [42]. Accordingly, the well-defined UV–visible absorption peak around 430 nm and the crystalline XRD pattern observed in the current study are consistent with earlier reports on biopolymer-mediated Cu and CuO nanoparticles [43,44]. Moreover, the mean crystallite size of the synthesized material was determined by Debye–Scherrer analysis of the characteristic XRD reflections, yielding a value of about 24.97 nm, confirming its nanocrystalline nature. It is important to distinguish this XRD-derived crystallite size from the physical particle size observed by microscopy, as crystallite size represents the dimensions of coherently diffracting crystalline domains, whereas an individual particle may comprise one or more crystallites.

The hemolysis assay demonstrated a concentration-dependent response. While hemolysis was relatively low at the lowest tested concentration, hemolytic activity increased with increasing Cu NP concentration, reaching 8.42% at 1000 µg/mL. Therefore, these findings do not support a general claim of biological safety; instead they indicate that the hemocompatibility of the synthesized Cu NPs is concentration-dependent.

Inflammation contributes significantly to diabetes pathogenesis by promoting insulin resistance and progressive β-cell dysfunction [45]. In vitro membrane stabilization assays, such as HRBC membrane protection and heat-induced hemolysis, are widely used as preliminary indicators of anti-inflammatory potential because they reflect the ability of test compounds to prevent membrane rupture and the subsequent release of inflammatory mediators. In the present study, Cu NPs exhibited concentration-dependent stabilization of human red blood cell membranes and inhibition of heat-induced hemolysis, indicating their capacity to protect biological membranes under inflammatory stress. Similar membrane-stabilizing effects have been reported for green-synthesized copper oxide nanoparticles, where reduced hemolysis was associated with attenuation of oxidative membrane damage and stabilization of phospholipid bilayers [46].

Postprandial hyperglycemia is mainly regulated by key digestive enzymes, including α-amylase and α-glucosidase, which hydrolyze complex carbohydrates into absorbable glucose [47]. Inhibiting these enzymes slows glucose consumption and reduces postprandial glycemic spikes [48]. In this study, chia-mediated Cu NPs showed pronounced, concentration-dependent inhibition of both α-amylase and α-glucosidase, consistent with earlier reports on biopolymer-functionalized CuO nanoparticles containing chitosan/PVA–CuO nanocomposites, which showed inhibition exceeding 80% at higher concentrations with distinct IC₅₀ values [49,50]. Beyond enzyme inhibition, enhanced glucose utilization signifies a critical complementary mechanism for glycemic control. The yeast glucose uptake assay, typically used as an initial screening model independent of insulin signaling, revealed a concentration-dependent increase in glucose uptake in yeast cells treated with Cu NPs. Similar improvements have been reported for green-synthesized copper-based nanoparticles derived from *Algerian propolis*, demonstrating that nanoparticle surface chemistry can affect glucose interaction and uptake dynamics [51]. Although in vitro assays provide valuable mechanistic insight, in vivo evaluation remains necessary to establish physiological relevance. The alloxan-induced diabetic mouse model is widely used to investigate anti-hyperglycemic agents, as alloxan selectively induces oxidative damage to pancreatic β-cells, resulting in persistent hyperglycemia [52]. In the current study, administration of Cu NPs resulted in a pronounced, dose-dependent reduction in blood glucose levels over a 30-day treatment period, with the highest dose (150 mg/kg) restoring glycemic levels close to those observed with metformin. These findings are consistent with proir in vivo studies on green-synthesized Cu nanoparticles in both alloxan- and streptozotocin-induced diabetic models [53–55]. The reduction in alloxan-induced hyperglycemia, together with the observed histological changes, may indicate preservation or recovery of pancreatic tissue. However, the present study did not directly measure oxidative stress, inflammatory signaling, or β-cell functional mechanisms [56,57]. The greater biological activity of the synthesized Cu NPs than chia seed polysaccharides alone suggests that nanoparticle formation enhanced the biological response. However, as chia-derived polysaccharides were used as reducing and stabilizing agents during green synthesis, a contribution from surface-associated polysaccharides to the observed activity cannot be completely excluded [58]. Therefore, the observed antidiabetic and anti-inflammatory effects are better interpreted as the activity of the complete green-synthesized nanoparticle formulation rather than being attributed exclusively to the copper component.

Diabetes is frequently associated with dysregulated lipid metabolism, which considerably contributes to cardiovascular risk [59]. In the current investigation, Cu NP treatment improved serum lipid parameters, including reductions in total cholesterol, triglycerides, and LDL levels, along with increased HDL concentrations. Histopathological analysis of pancreatic and hepatic tissues further supported the biochemical findings, revealing restoration of islet architecture and reduced hepatic necrosis in Cu NP-treated groups. Similar tissue-protective properties have been reported in earlier CuO NP studies and are likely attributable to the combined anti-inflammatory, antioxidant, and metabolic regulatory effects of Cu NPs stabilized with biocompatible polysaccharides [53,60].

The present study has some limitations. The study did not determine hydrodynamic size distribution, surface charge, precise primary particle size, copper oxidation state, or thermal characteristics using DLS/PDI, zeta-potential, TEM, EDS/XPS, and TGA/DSC, respectively. Furthermore, the present study did not include detailed mechanistic analysis or long-term evaluation in various diabetic models. Further studies with detailed physicochemical characterization and mechanistic studies are warranted to support these findings and their translational relevance. Furthermore, the absence of a copper-precursor-only control limits definitive separation of the contribution of the copper component from that of the complete polysaccharide-associated nanoparticle formulation.

## 5. Conclusion

The present study demonstrated the antidiabetic and anti-inflammatory potential of chia seed polysaccharide-mediated Cu NPs, as evidenced by experimentally observed enzyme-inhibitory activity, enhanced glucose uptake, reduced blood glucose levels, improved serum lipid parameters, and restoration of pancreatic and hepatic tissue architecture. These findings support the biological activity of the green-synthesized Cu NPs under the experimental conditions investigated. However, the molecular mechanisms underlying these effects, including possible involvement of oxidative stress and inflammatory signaling pathways, were not directly evaluated in the present study and therefore require further investigation. Overall, the findings support further investigation of chia seed polysaccharide-mediated Cu NPs, but additional mechanistic, physicochemical, and safety studies are needed to establish their potential biological applications.
